# Influence of Bonding Temperature on Microstructure and Mechanical Properties of AZ31/Zn/Sn/5083 Diffusion Joint

**DOI:** 10.3390/ma17246110

**Published:** 2024-12-13

**Authors:** Tianbao Tan, Yangyang Guo, Gang Chen, Zijun Rong, Houhong Pan

**Affiliations:** 1 Key Laboratory of Advanced Technologies of Materials, Ministry of Education China, School of Materials Science and Engineering, Southwest Jiaotong University, Chengdu 610031, China; 2 Northwest Institute for Non-Ferrous Metal Research, Xi’an 710016, China; 15108226903@163.com

**Keywords:** diffusion bonding, microstructure, mechanical properties, magnesium alloy, aluminum alloy, Zn/Sn composite interlayer

## Abstract

Diffusion bonding with an interlayer is considered an effective means of obtaining Mg/Al dissimilar alloy joints. However, at low temperatures, it is often impossible to simultaneously achieve joints between the interlayer and Mg/Al under the same bonding parameters. For this reason, the interlayer is usually prefabricated on the substrate, followed by conducting diffusion bonding. Due to the higher diffusion rate of atoms in the liquid phase compared to atoms in the solid phase, creating a liquid phase field in diffusion bonding to reduce diffusion resistance and thus omitting the step of prefabricating the interlayer is a feasible approach. In this study, solid-state diffusion bonding and TLP (transient liquid phase) diffusion bonding were combined. The low-temperature diffusion bonding of the Mg/Al alloy was achieved under the same parameters using a Zn/Sn composite interlayer, utilizing the formation of a Zn-Sn eutectic liquid phase and the complete melting of Sn during heating without requiring a prefabricated interlayer. Unlike conventional composite interlayers used in diffusion bonding, the Sn layer of the Zn/Sn composite interlayer completely melts into liquid and is squeezed out of the bonding interface at the bonding temperature. The Mg/Zn interface was bonded by solid-state diffusion bonding, while the Al/Zn interface was joined through TLP diffusion bonding. Research on the bonding temperature showed that the bonding temperature range was narrow and that variation in the bonding temperature had a significant impact on the microstructure of the joints.

## 1. Introduction

Magnesium (Mg) alloys with excellent specific strength and electromagnetic shielding properties are regarded as new lightweight structural materials. Aluminum (Al) alloys with low density and excellent corrosion resistance have been widely used in the aircraft and automobile industries. High-quality joints composed of Mg and Al are of great significance for reducing the weight of structures and expanding the application ranges of these two metals. However, regardless of the welding method used, direct welding of Mg and Al results in the formation of Mg-Al intermetallic compounds (IMCs) with high brittleness in the joint, reducing its performance [1,2,3,4,5,6,7,8,9,10]. Diffusion bonding with an interlayer is a suitable welding process to join dissimilar materials [11,12]. Due to limiting of the eutectic point temperature between Mg and the interlayer and between Al and the interlayer, the bonding temperature of Mg/Al diffusion bonding remains low. When the interlayer material is directly inserted between Mg and Al, it is difficult to join Mg and Al to the interlayer simultaneously under the same bonding conditions [13]. Thus, some scholars have used different methods to add interlayer material for the diffusion bonding of Mg/Al. Jie, J.C. et al. [14] prepared Al/Mg bimetal via diffusion bonding using Ni-based conversion plating and Ni-P electroless plating. Yin, F.X. et al. [15] investigated the diffusion bonding of Mg and Al with a Ni interlayer prepared by plasma spraying technology. Wang, Y.Y. et al. [16] carried out the diffusion bonding of Mg1 and 1060 Al alloy with an Ag interlayer prepared by magnetron sputtering physical vapor deposition (PVD). Liu, L.M. et al. [17] plated Zn-4Al-0.1Ce alloy on the surface of 6061 Al alloy using hot-dip plating (HDP), then carried out AZ31B/Zn-4Al-0.1Ce/6061 diffusion bonding at a bonding temperature of 365 °C. Shakeri, H. et al. [18] compared Ag foil and PVD Ag film as interlayers in the diffusion bonding of AZ31 Mg alloy and 5083 Al alloy at a bonding temperature of 470 °C and found that the joints in the PVD Ag film interlayer had a higher bonding strength. Habisch, S. et al. [19] deposited a 2-μm Ti film through PVD on the surface of Al alloy as an interlayer to join AZ31B to AA7020 through diffusion bonding, with bonding conditions of 415 °C–8.5 MPa–60 min. Zhang, J. et al. [20] prepared a 2-μm Al film through magnetron sputtering PVD on the surface of pure Mg, then pure Mg with PVD Al film was bonded to pure Al by diffusion bonding using pure Ni as an interlayer. Wang, Y. et al. [21] used HDP Al-Zn alloy plating as an interlayer for the diffusion bonding of AZ31 Mg alloy to AA6111 Al alloy. Nimal, R.J.G.R. et al. [22] coated Al on both AZ80 Mg alloy and 7075 Al alloy, then carried out diffusion bonding of the AZ80 Mg alloy and 7075 Al alloy.

The above methods require prefabrication of the interlayer by plating, spraying, PVD, or HDP on one or both base materials before diffusion bonding, which increases the complexity of the bonding process. In this work, a Zn/Sn composite interlayer is used. By forming a Zn-Sn eutectic liquid phase and completely melting Sn during the bonding process, diffusion bonding of Mg/Al can be achieved without a prefabrication process. During bonding, Mg/Zn is bonded by solid-state diffusion bonding, while Al/Zn is joined by TLP (transient liquid phase) diffusion bonding. This paper focuses on the microstructure and mechanical properties of the bonded joints at different bonding temperatures. SEM and EDS were used to investigate the bonding interface and the shear-fractured surface. The shear test and microhardness test were used to examine the strength and hardness of the joints.

## 2. Experimental Procedure

**Figure 1:**
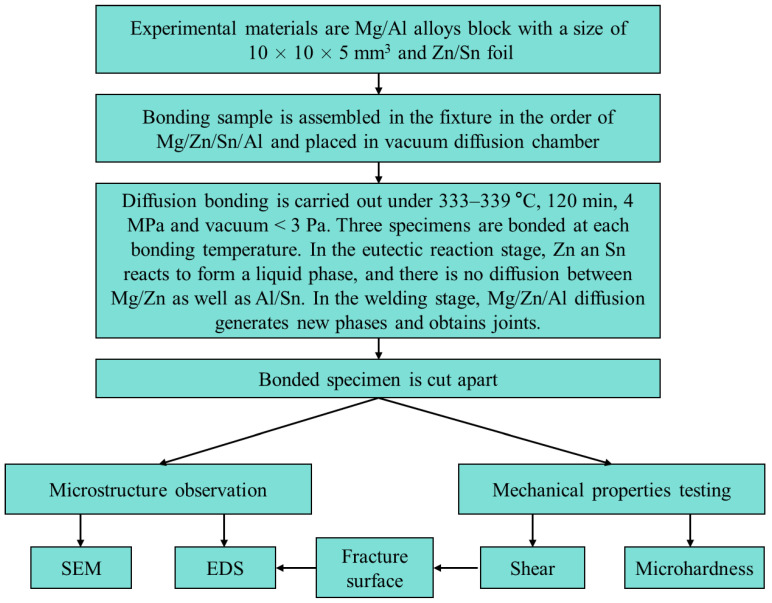
Flowchart of the research.

**Figure 2:**
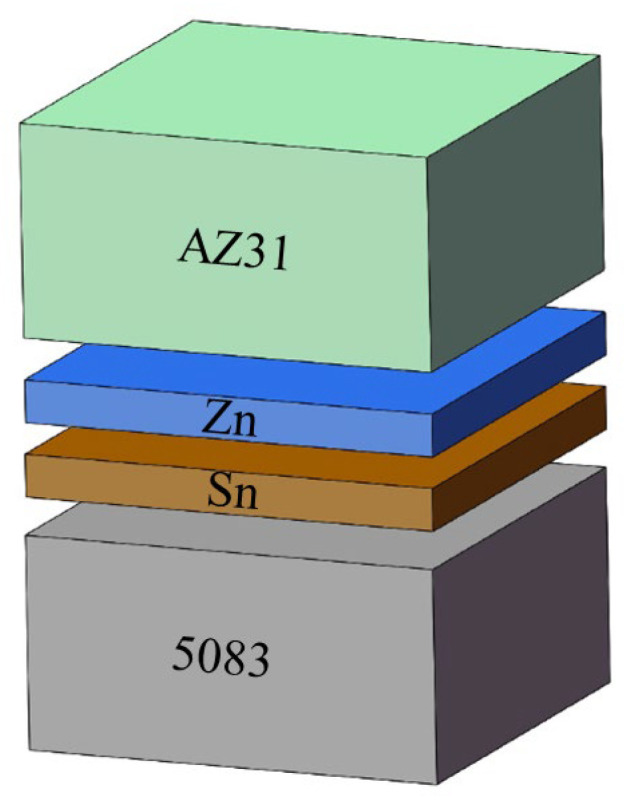
Assembly schematic of the diffusion couple.

**Figure 3:**
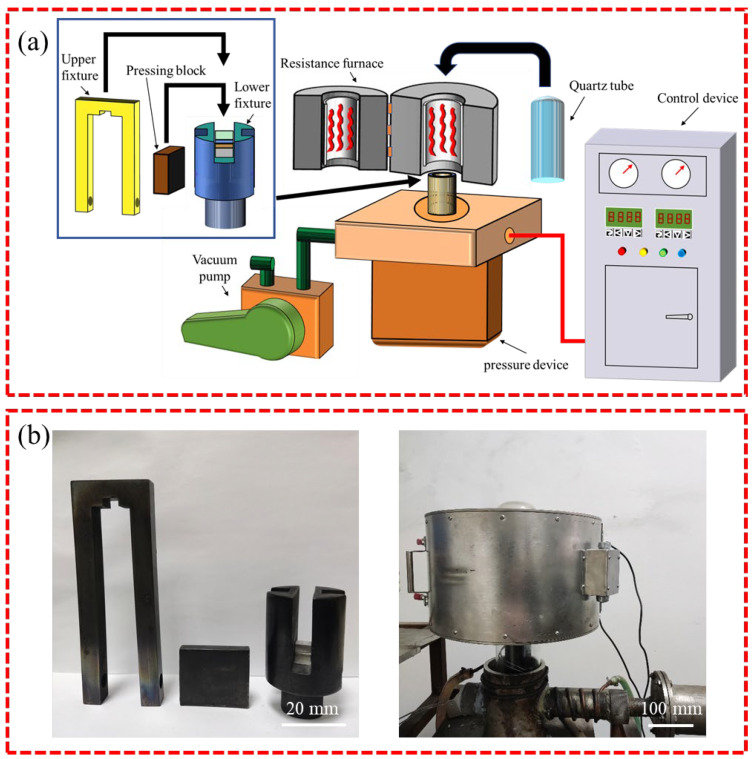
(**a**) Schematic diagram of bonding fixture and diffusion bonding equipment, and (**b**) Physical images of bonding fixture and resistance vacuum furnace.

**Figure 4:**
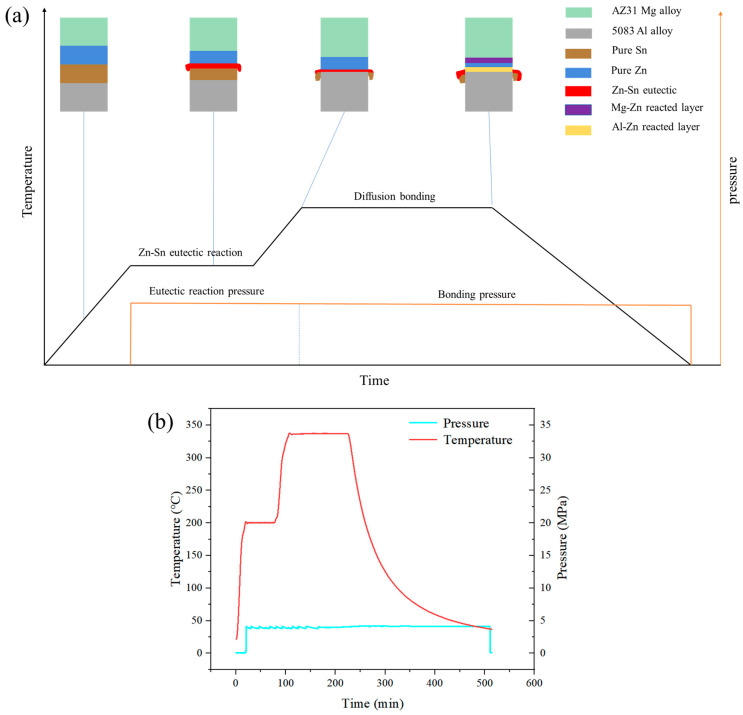
Diffusion procedure: (**a**) Schematic, and (**b**) Real-time.

**Figure 5:**
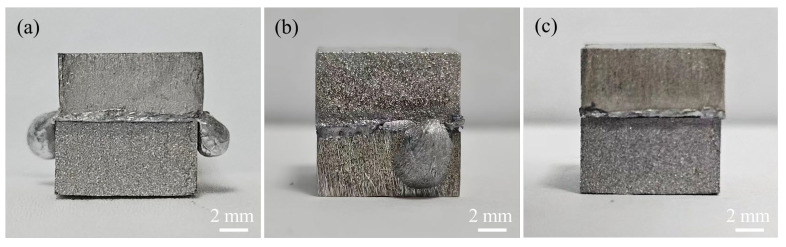
The bonded specimens: (**a**) 333 °C, (**b**) 336 °C, and (**c**) 339 °C.

A research flowchart is shown in Figure 1. Table 1 shows the chemical compositions of the AZ31 Mg alloy and 5083 Al alloy used in this experiment. The samples of both base materials were machined to the dimensions 10 × 10 × 5 mm^3^. The interlayer metals were a 600 μm-thick layer of 99.8% pure Sn and a 120 μm-thick layer of 99.9% pure Zn. The Zn layer was the main interlayer for isolating Mg and Al, while the Sn layer was an auxiliary interlayer that formed a Zn-Sn eutectic liquid with Zn. Before bonding, the welding surfaces of the samples were ground with SiC papers until grit 2000 and polished with 2.5 μm of diamond paste. Then, the samples were ultrasonically cleaned in acetone for 15 min. The diffusion couples were assembled in the order shown in Figure 2 and then put into the joining fixture. Diffusion bonding was carried out in a self-made vacuum diffusion furnace. A schematic diagram of the bonding fixture and diffusion bonding equipment, as well as physical images of the bonding fixture and resistance vacuum furnace, are shown in Figure 3. The heating rate was 15 °C/min. The pressure device pulled down the upper fixture to apply bonding pressure to the bonded sample. A complete bonding process included two stages: Zn-Sn eutectic reaction and diffusion bonding. A schematic of the diffusion procedure is shown in Figure 4, and the bonding process parameters are listed in Table 2. At the eutectic reaction temperature between the Zn-Sn eutectic point (Teu_Zn-Sn_: 195.5 °C) and the Sn melting point (Tm_Sn_: 231.93 °C), Zn-Sn eutectic liquid was formed at the Zn/Sn interface, and excessive Zn-Sn eutectic liquid was squeezed out of the Zn/Sn interface by the eutectic reaction pressure. When the temperature was raised to above the Sn melting point, the Sn was completely melted into a liquid state and was squeezed out of the Al/Zn interface by the bonding pressure. At the bonding temperature, Al/Zn was joined by TLP diffusion bonding, while Mg/Zn was joined by solid-state diffusion bonding. After bonding, the samples were cooled in a furnace. Figure 5 shows the bonded specimens. At each bonding temperature, a total of three specimens were bonded.

**Figure 6:**
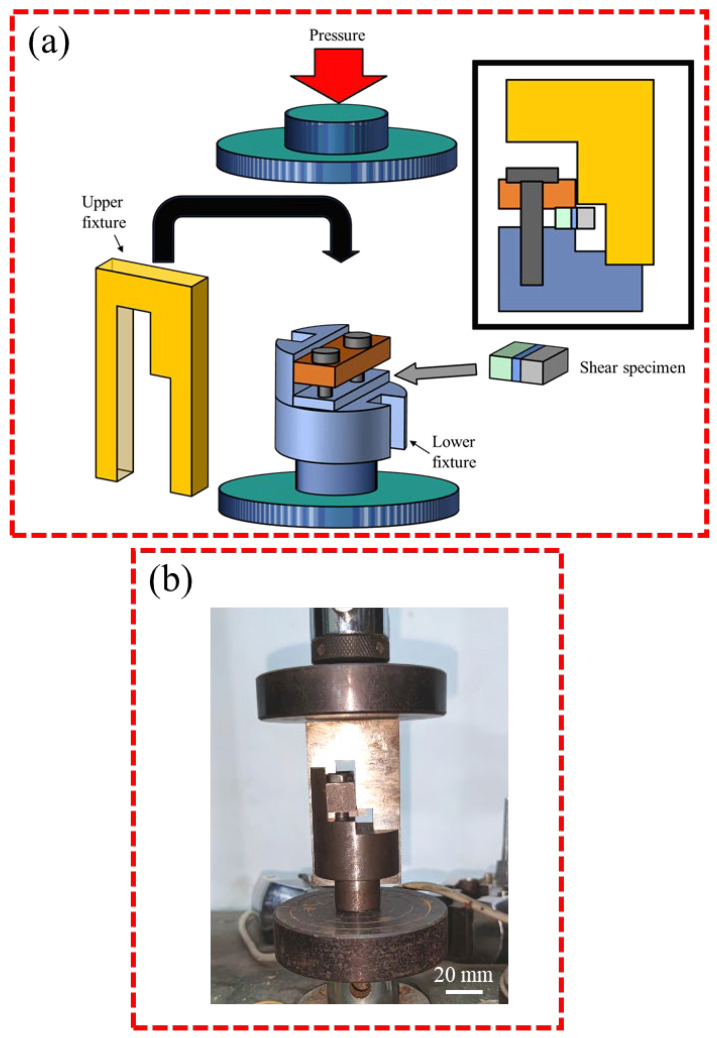
(**a**) Schematic diagram, and (**b**) Practical picture of the shear test.

**Figure 7:**
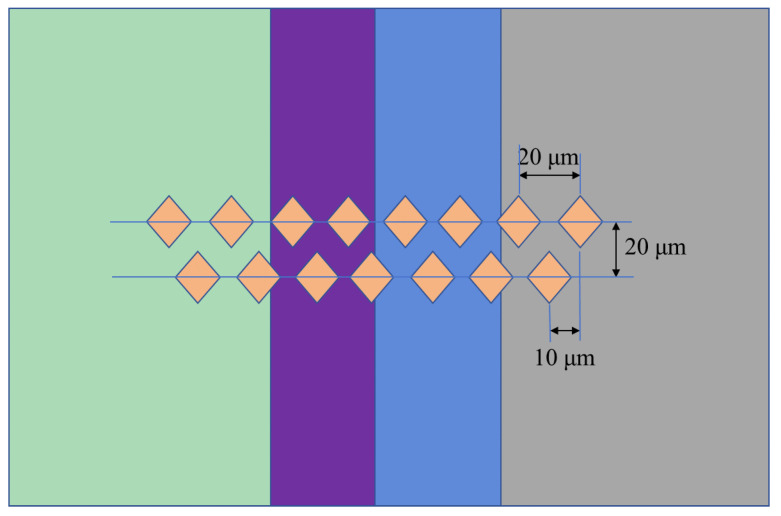
Schematic diagram of the indentation of hardness test.

All welded specimens were cut into two pieces in the perpendicular direction of the bonding interface by wire-electrode cutting. One piece was used to shear and analyze the fracture surface, and the other was used for the interfacial microstructure analysis and hardness test. The cutting surfaces of the samples were ground and polished in the traditional way and then etched using a mixed solution of 4.2 g picric acid, 10 mL of acetic acid, 10 mL of distilled water, and 100 mL of ethanol. The microstructures of the joints were observed by scanning electron microscope (SEM, JSM7800F, JEOL, Tokyo, Japan). Energy-dispersive spectroscopy (EDS, X-Max 80, Oxford, UK) was used to analyze the concentration of the chemical elements. The shear specimens were ground and shaped to dimensions of 8 mm × 8 mm × 3.5 mm and then fixed to the shear fixture. A schematic diagram and practical picture of the shear test are shown in Figure 6. The shear test was carried out on the universal testing machine (CMT5105, MTS, Beijing, China) with a crosshead speed of 0.5 mm/min. There were three shear specimens at each bonding temperature, and the shear strength was their average value. Three microhardness measurements were conducted on a selected specimen. The hardness value was the average of three tests. The microhardness was measured by Vickers hardness tester (DHV-1000ZTEST, Qichen, Binzhou, China) and employed a non-standard method. The microhardness test adopted the double-row indentation method with a spacing of 20 μm. The measurement step distance was determined to be 20 μm based on the indentation size of the softest AZ31 Mg alloy. The dislocation of indentation points between the two rows was 10 μm. The test load was 10 gf, and the residence time under load was 15 s. Figure 7 is the schematic diagram of the hardness test. The hardness point distance after the combination of two rows of data was 10 μm. The selected shear fracture surfaces were examined by SEM and X-ray diffraction (XRD, Empyrean, Malvern Panalytical, Almelo, The Netherlands).

## 3. Results and Discussion

### 3.1. Microstructure of the Joint Bonded at 333 °C

**Figure 8:**
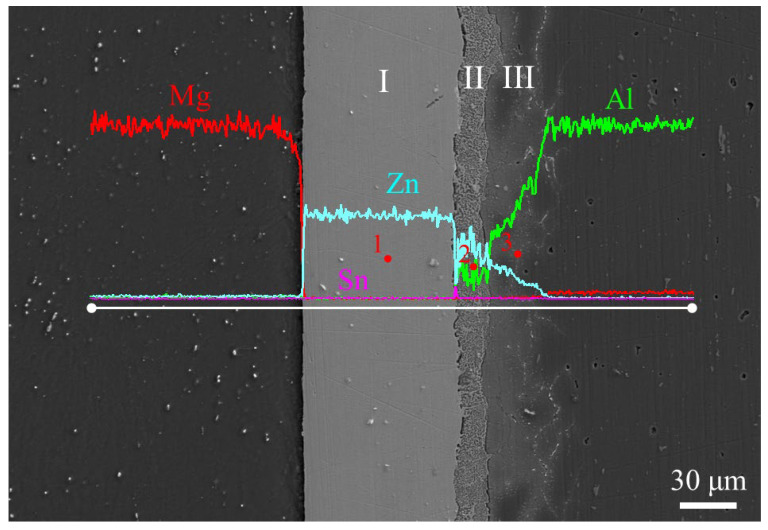
SEM, EDS line-scanning results across the bonding interface and EDS point-analysis position of the joint bonded at 333 °C.

Figure 8 shows the SEM and EDS line-scan results of the joint bonded at 333 °C. The EDS point-analysis data and the possible phases for each point in Figure 8 are listed in Table 3. As seen in Figure 8, the layered characteristics of the bonding interface were obvious. The interface of the joint can be divided into three layers. The concentration of elements in Layer 1 was unchanged, and the Zn content was as high as 95.7 at. % (Point 1 in Figure 8). Therefore, Layer I was regarded as the residual Zn layer. Due to the low diffusion temperature, the mutual diffusion between Mg and Zn was insufficient, and no obvious diffusion reaction layer was formed at the Mg/Zn interface. The concentrations of Al and Zn in Layer II fluctuated within a certain range. It is known that the solid solubility of Al in Zn is low. It has been reported [23,24] that after the Zn(Al) solid solution reaches saturation, the Al-Zn eutectoid phase (α + η) is formed. Thus, Layer II was considered to be an α + η layer. Layer III showed an increase in Al concentration and a decrease in Zn concentration from the Zn side to the 5083 Al alloy side, indicating that Layer III was an Al(Zn) solid solution layer. In our previous study [13], it was difficult to realize the effective joining of Al/Zn by solid-state diffusion bonding at 333 °C. In this experiment, the Zn-Sn eutectic liquid was used to join Al to Zn by TLP diffusion bonding, resulting in the formation of a double-layer structure of α + η and Al(Zn) solid solution between Zn and the 5083 Al alloy. Compared with Al/Zn solid-state diffusion bonding [13], the bonding temperature of Al/Zn TLP diffusion bonding by the Zn-Sn eutectic liquid phase was reduced by 30 °C.

### 3.2. Microstructure of the Joint Bonded at 336 °C

**Figure 9:**
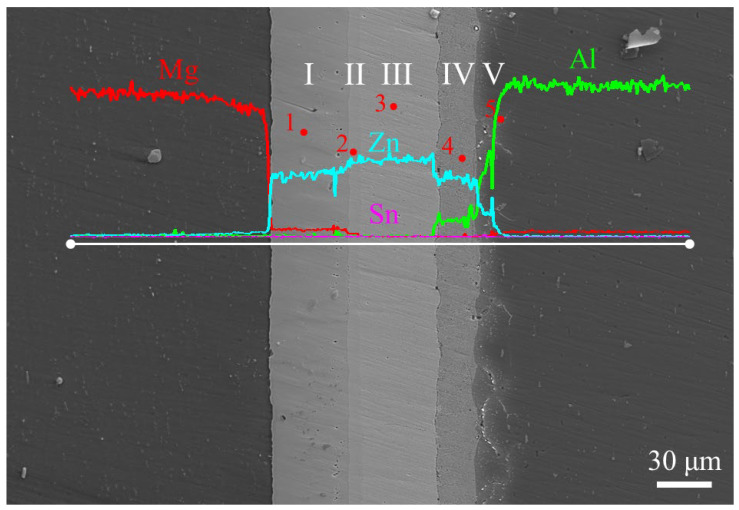
SEM and EDS line-scanning results across the bonding interface and EDS point-analysis position of the joint bonded at 336 °C.

SEM and EDS line-scanning results of the joint bonded at 336 °C are shown in Figure 9. The EDS point-analysis data and the possible phases for each point in Figure 9 are listed in Table 4. According to Figure 9, the joint was divided into five layers: I, II, III, IV, and V. Compared with the joint at 333 °C, the difference was that two diffusion reaction layers were formed between the AZ31 Mg alloy and the Zn interlayer at 336 °C. It can be seen from Figure 9 that both Layer I and Layer II had constant concentrations of Mg and Zn, and the content of Al and Sn in Points 1 and point 2 was low. The atomic ratios of Mg and Zn in Layer I and Layer II were about 1:2 and 2:11, respectively. Combining the Mg-Zn binary phase diagram [25], Layer I and Layer II were identified as the MgZn_2_ and Mg_2_Zn_11_ layers, respectively. The EDS line-scanning and EDS point analysis of Layer III, Layer IV, and Layer V at this joint were similar to Layer I, Layer II, and Layer III in Figure 8, respectively. Therefore, Layer III, Layer IV, and Layer V of this joint were considered to be the residual Zn layer, α + η layer, and Al(Zn) solid solution layer, respectively.

**Figure 10:**
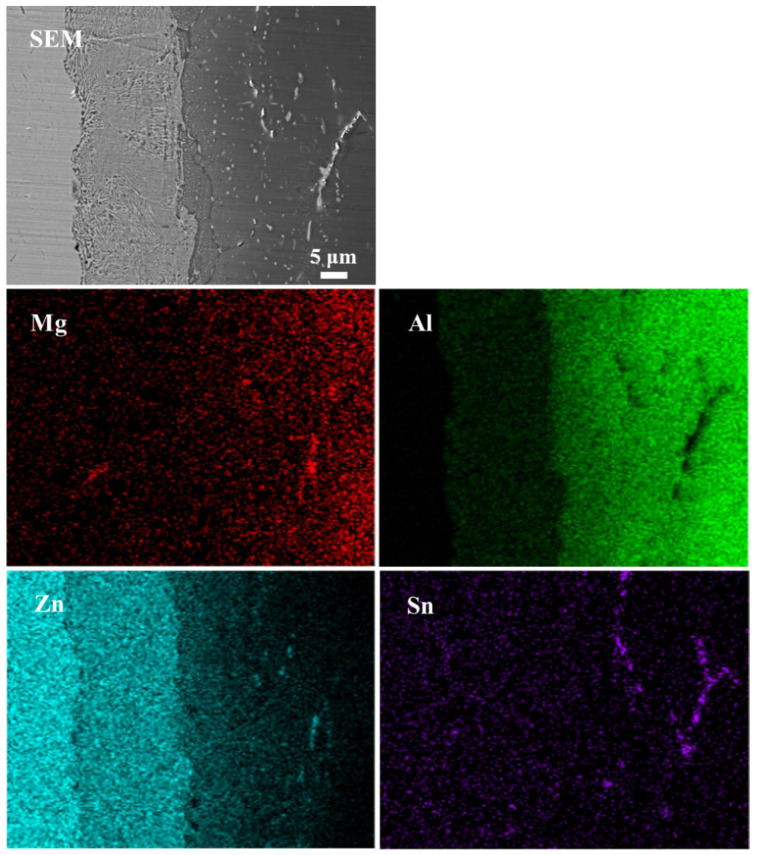
EDS mapping analysis results at the Al/Zn interface of the joint bonded at 336 °C.

Figure 10 shows the EDS mapping analysis results of the Al/Zn interface of the joint bonded at the bonding temperature of 336 °C. It can be seen from Figure 10 that no aggregation of Sn was found in the Al/Zn diffusion reaction zone. In addition, Sn was distributed independently in the 5083 Al alloy in a linear shape, which indicated that Sn diffused along grain boundaries in the 5083 Al alloy and formed a “network” structure of Sn segregation.

### 3.3. Microstructure of Joint Bonded at 339 °C

**Figure 11:**
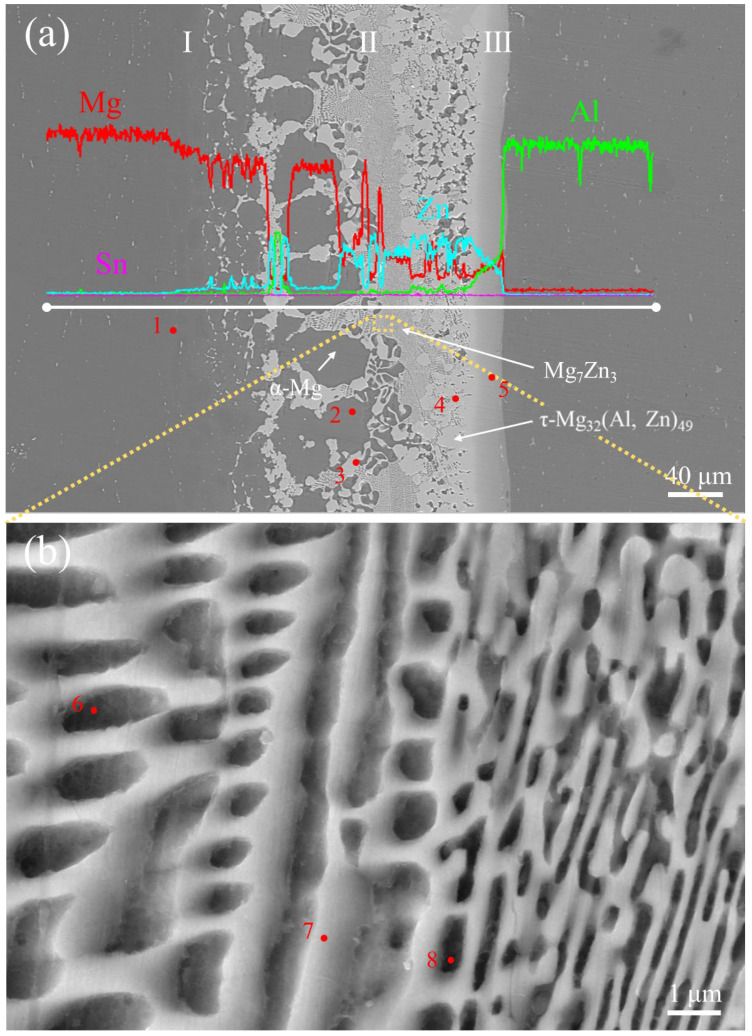
SEM and EDS line-scanning results across the bond interface and EDS point-analysis position of (**a**) the joint bonded at 339 °C and (**b**) Magnification of the dark gray phase zone.

Figure 11 is the SEM image and EDS line-scanning result of the joint bonded at a bonding temperature of 339 °C. The EDS point-analysis results and the possible phases in different positions of this joint are shown in Table 5. As shown in Figure 11, the microstructure of the joint was similar to the microstructure of brazing. Compared with the joints at 333 °C and 336 °C, the difference in the microstructure at 339 °C was obvious. The joint can be divided into three regions, marked as Ⅰ, Ⅱ, and III. In Region Ⅰ, Al concentration increased, and Zn concentration decreased from the AZ31 Mg alloy side to the 5083 Al alloy side. Therefore, Region I was identified as the α-Mg solid solution region. Region Ⅱ was composed of a dark phase, a dark gray phase, and a light gray phase. The dark phase was mainly distributed on the AZ31 Mg alloy side, and the light gray phase was mainly distributed on the 5083 Mg alloy side, while the dark gray phase was distributed in the middle part of this region. According to the composition of Point 3 (15.2 at. % Al, 44.8 at. % Mg, and 40.0 at. % Zn) and Point 4 (16.3 at. % Al, 43.8 at. % Mg, 39.8 at. % Zn, and 0.1 at. % Sn), the light gray phase was identified as the τ-Mg_32_(Al, Zn)_49_ phase [26]. The dark phase was made up of 93.9 at. % Mg, 3.1 at. % Zn, 2.6 at. % Al, and 0.4 at. % Sn (Point 2), which was identified as the α-Mg solid solution phase. The dark gray phase presented a grid structure, as shown in the lower-left corner of Figure 11. EDS point analysis at Points 6–8 indicated that its components were 3.3–6.1 at. % Al, 67.3–76.6 at. % Mg, 19.8–26.4 at. % Zn, and 0.2–0.4 at. % Sn and the atomic ratio of Mg and Zn was close to 7:3. According to the Mg-Zn binary phase diagram [25], the dark gray phase was identified as Mg_7_Zn_3_. Therefore, Region II was regarded as the α-Mg + Mg_7_Zn_3_ + τ mixing region. It is worth noting that the Mg_7_Zn_3_ phase is a metastable phase that can easily be transformed to α-Mg + Mg-Zn, resulting in the coexistence of Mg_7_Zn_3_, Mg-Zn, and α-Mg. In Region III, the concentration of Al and Mg increased, while Zn concentration decreased from the AZ31 Mg alloy side to the 5083 Al alloy side, which indicated that Region III was made up of Al-Mg-Zn ternary solid solution. The Mg-Al IMCs, e.g., Mg_2_Al_3_ and Mg_17_Al_12_, were not detected in the joint due to the higher activation energy required for the Mg-Al reaction compared with the Mg-Zn-Al reaction [27].

### 3.4. Microhardness of the Joints

**Figure 12:**
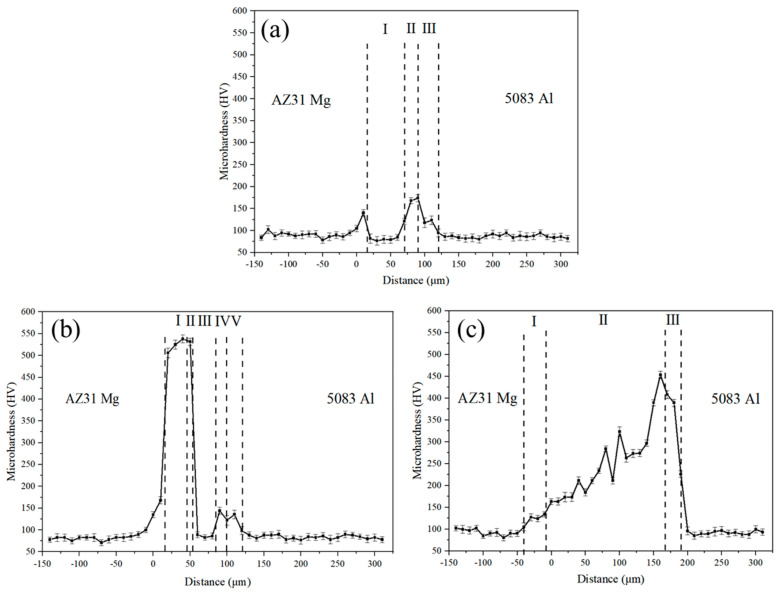
Microhardness profiles across the interface of the joints bonded at different bonding temperatures: (**a**) 333 °C, (**b**) 336 °C and (**c**) 339 °C.

Microhardness across the bonding interface of the joints bonded at the different bonding temperatures is presented in Figure 12. The average hardness values of the base materials and the interlayer material were about 84 HV (5083 Al), 80 HV (AZ31 Mg), and 74 HV (pure Zn), respectively. When the bonding temperature was 333 °C, it can be seen from Figure 12a that the maximum hardness value of the joint was 175 HV obtained at the Al/Zn interface due to the formation of the α + η phase. At a bonding temperature of 336 °C, as shown in Figure 12b, the maximum hardness value of the joint increased markedly to 538 HV at the Mg/Zn interface, which is related to the formation of Mg_2_Zn_11_ IMCs. As the bonding temperature further increased to 339 °C, the joint had a maximum hardness of 450 HV located on the Al side of the α-Mg + Mg_7_Zn_3_ + τ mixing region (see Figure 12c), which was attributed to the formation of the τ phase. However, the hardness value of the τ phase was lower than that of Mg_2_Zn_11_ IMCs. The gradual increase in hardness from the AZ31 Mg side to the 5083 Al side in Figure 12c corresponds to the gradual transformation process of the microstructure from α–Mg to Mg_7_Zn_3_ and the τ phase.

### 3.5. Shear Strength of the Joints

**Figure 13:**
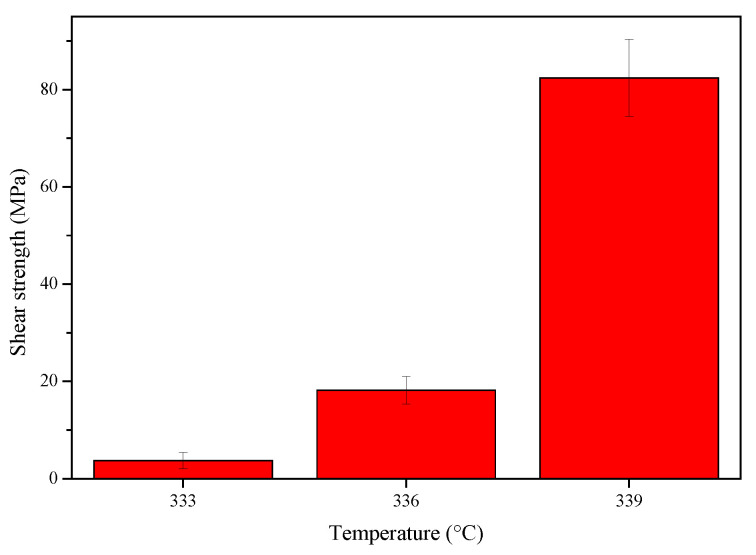
Shear strength of the Mg/Zn/Sn/Al joints bonded at different bonding temperatures.

Figure 13 shows the shear strength of the Mg/Zn/Sn/Al joints bonded at the different bonding temperatures. It can be seen from Figure 13 that the shear strength of the joint increased with the increase of the bonding temperature. When the bonding temperature was 333 °C, the shear strength of the joint was only 2.1 MPa due to insufficient atomic diffusion at the Mg/Zn interface. When the bonding temperature rose to 339 °C, the joint had a maximum shear strength value of 76.8 MPa.

### 3.6. Shear Fracture Behavior of the Joints

**Figure 14:**
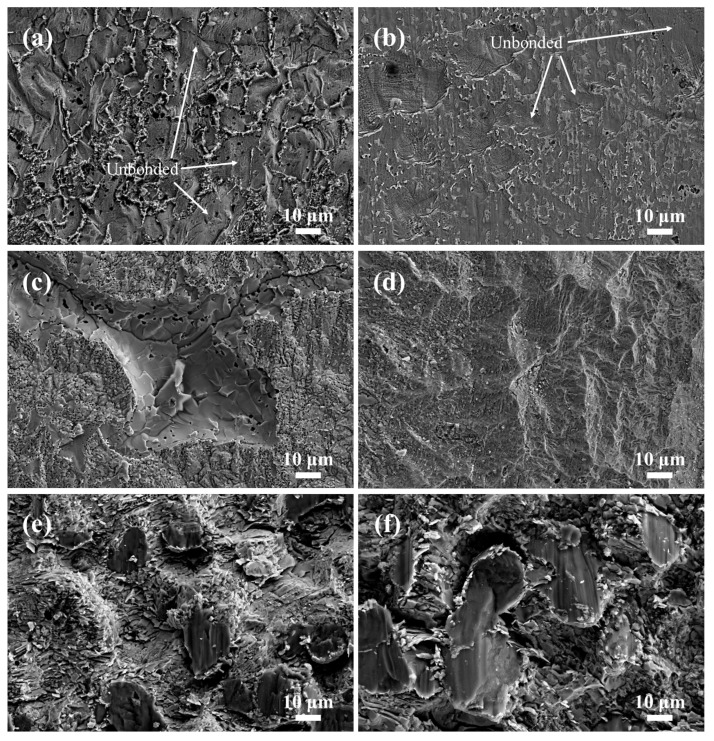
SEM images of shear fracture surfaces of Mg/Zn/Sn/Al joints with the Zn-Sn eutectic reaction time of 60 min at different bonding temperatures: (**a**) 333 °C at Mg side, (**b**) 333 °C at Al side, (**c**) 336 °C at Mg side, (**d**) 336 °C at Al side, (**e**) 339 °C at Mg side, and (**f**) 339 °C at Al side.

**Figure 15:**
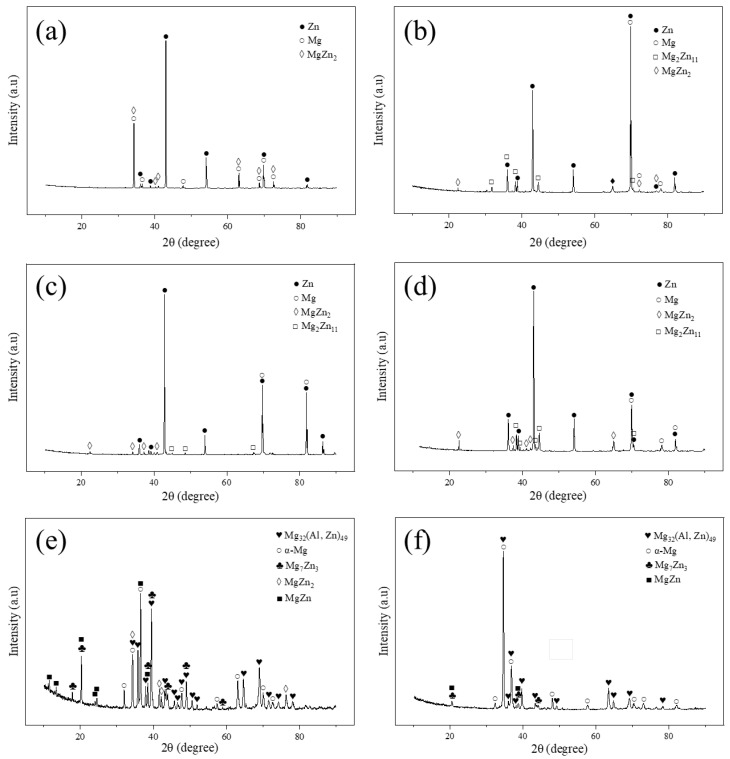
XRD patterns of shear fracture surfaces of Mg/Zn/Sn/Al joints with the Zn-Sn eutectic reaction time of 60 min at different bonding temperatures: (**a**) 333 °C at Mg side, (**b**) 333 °C at Al side, (**c**) 336 °C at Mg side, (**d**) 336 °C at Al side, (**e**) 339 °C at Mg side, and (**f**) 339 °C at Al side.

To further study the shear fracture mode and the shear fracture location of the Mg/Zn/Sn/Al joints, the shear fracture morphology of the joints and the phase of the shear fracture surface were analyzed, and the results are presented in Figure 14 and Figure 15. The joint bonded at 333 °C with low shear strength showed planar unbonded regions in the shear fracture surface (Figure 14a,b). Strong Zn and Mg diffraction peaks can be clearly observed in Figure 15a,b, indicating the shear fracture located at the Mg/Zn interface as a result of its insufficient diffusion. Figure 14c,d display the characteristics of cleavage morphology and a small amount little dimple morphology (right side of Figure 14d) for the joint bonded at 336 °C, which showed that the shear fracture of the joint was mainly brittle. According to the XRD patterns in Figure 15c,d, there were Zn and Mg-Zn IMCs on both sides of the shear fracture surface, implying that the shear fracture was located between the Zn layer and the Mg-Zn IMC layer. In Figure 14e,f, the shear fracture surface of the joint bonded at 339 °C was characterized by cleavage morphology, which was of typical brittle fracture. As shown in Figure 15e,f, α-Mg, τ, and Mg_7_Zn_3_ were detected on both sides of the shear fracture surface, suggesting that the shear fracture position was in α-Mg + Mg_7_Zn_3_ + τ mixing region.

## 4. Conclusions

The influence of the bonding temperature on the microstructure and mechanical properties of the AZ31/Zn/Sn/5583 diffusion joint was investigated. According to the findings and limitations of this research, the conclusion is shown as follows.

(1) The bonding temperature varied, and the microstructure of the joints changed significantly. The bonding temperatures were 333 °C, 336 °C, and 339 °C, and the structures of the joints were AZ31/Zn/α + η/Al(Zn)/5083, AZ31/Mg(Zn)/MgZn_2_/Mg_2_Zn_11_/Zn/α + η/Al(Zn)/5083, and AZ31/Mg(Zn)/α-Mg + Mg_7_Zn_3_ + τ/Mg-Al-Zn solid solution/5083, respectively.
(2) The maximum hardness value of the joints was 538 HV at the Mg-Zn IMC layer of the joint at 336 °C, and the highest shear strength of 78.3 MPa was achieved for the joint at 339 °C.
(3) The shear fracture mode of all joints was brittle fracture. For the joints at 333 °C, 336 °C, and 339 °C, the shear fracture positions were located at the Mg/Zn interface, the Mg-Zn IMC layer, and the α-Mg + Mg7Zn3 + τ mixing region, respectively.
(4) The formation of the Zn-Sn eutectic liquid phase reduced the Al/Zn bonding temperature, achieving a low-temperature diffusion bonding of AZ31/5083. However, limited by the diffusion and reaction of the Mg/Zn interface, the bonding temperature range was narrow, and a slight change in the bonding temperature had a significant effect on the joint structure. Therefore, the actual application requires the equipment to have a high control accuracy of temperature.
(5) The structure and thickness of each layer had a significant impact on the strength of the joint. Therefore, further research is needed using collaborative adjustment of the eutectic reaction time and the bonding time to obtain greater strength of the joint.

## Figures and Tables

**Table 1:** Chemical compositions of the materials used in this experience (wt. %).

Material	Mg	Zn	Cr	Mn	Ti	Ca	Al	Bi	Si
AZ31	Bal	1.03	—	0.22	—	0.04	2.99	—	0.08
5083	4.51	0.03	0.12	0.58	0.02	—	Bal	—	0.03

**Table 2:** Process parameters used in this experiment.

Sn-Zn Eutectic Reaction			Diffusion Bonding			Vacuum Degree (Pa)
Temperature (°C)	Time (min)	Pressure (MPa)	Temperature (°C)	Time (min)	Pressure (MPa)	
200	60	4	333, 336, 339	120	4	<3 × 10^−1^

**Table 3:** EDS point-analysis results at different positions in the joint (marked in Figure 8).

Point Number	Composition (at. %)				Phase Identity
	Al	Mg	Zn	Sn	
1	2.1	2.2	95.7	0	Zn
2	30.2	1.2	68.3	0.3	α + η
3	85.6	0.8	13.0	0.6	Al(Zn)

**Table 4:** EDS point-analysis results at different positions in the joint (marked in Figure 9).

Point Number	Composition (at. %)				Phase Identity
	Al	Mg	Zn	Sn	
1	1.7	32.8	65.4	0.1	MgZn_2_
2	1.3	16.0	82.6	0.1	Mg_2_Zn_11_
3	1.7	0.9	97.3	0.1	Zn
4	41.6	2.1	56.3	—	α + η
5	86.8	0.3	12.8	0.1	Al(Zn)

**Table 5:** EDS point-analysis results at different positions in the joint (marked in Figure 11).

Point Number	Composition (at. %)				Phase Identity
	Al	Mg	Zn	Sn	
1	2.5	94.5	3.0	0	α-Mg
2	2.6	93.9	3.1	0.4	α-Mg
3	15.2	44.8	40.0	0	τ-Mg_32_(Al, Zn)_49_
4	16.3	43.8	39.8	0.1	τ-Mg_32_(Al, Zn)_49_
5	39.0	39.8	21.2	0	Al-Mg-Zn solid solution
6	3.8	71.1	24.7	0.4	Mg_7_Zn_3_
7	3.2	76.6	19.8	0.4	Mg_7_Zn_3_
8	6.1	67.3	26.4	0.2	Mg_7_Zn_3_

## Data Availability

The original contributions presented in this study are included in the article. Further inquiries can be directed to the corresponding author.
